# Wall Shear Stress Effects on Endothelial-Endothelial and Endothelial-Smooth Muscle Cell Interactions in Tissue Engineered Models of the Vascular Wall

**DOI:** 10.1371/journal.pone.0088304

**Published:** 2014-02-10

**Authors:** Dalit Shav, Ruth Gotlieb, Uri Zaretsky, David Elad, Shmuel Einav

**Affiliations:** Department of Biomedical Engineering, Tel Aviv University, Tel Aviv, Israel; UAE University, Faculty of Medicine & Health Sciences, United Arab Emirates

## Abstract

Vascular functions are affected by wall shear stresses (WSS) applied on the endothelial cells (EC), as well as by the interactions of the EC with the adjacent smooth muscle cells (SMC). The present study was designed to investigate the effects of WSS on the endothelial interactions with its surroundings. For this purpose we developed and constructed two co-culture models of EC and SMC, and compared their response to that of a single monolayer of cultured EC. In one co-culture model the EC were cultured on the SMC, whereas in the other model the EC and SMC were cultured on the opposite sides of a membrane. We studied EC-matrix interactions through focal adhesion kinase morphology, EC-EC interactions through VE-Cadherin expression and morphology, and EC-SMC interactions through the expression of Cx43 and Cx37. In the absence of WSS the SMC presence reduced EC-EC connectivity but produced EC-SMC connections using both connexins. The exposure to WSS produced discontinuity in the EC-EC connections, with a weaker effect in the co-culture models. In the EC monolayer, WSS exposure (12 and 4 dyne/cm^2^ for 30 min) increased the EC-EC interaction using both connexins. WSS exposure of 12 dyne/cm^2^ did not affect the EC-SMC interactions, whereas WSS of 4 dyne/cm^2^ elevated the amount of Cx43 and reduced the amount of Cx37, with a different magnitude between the models. The reduced endothelium connectivity suggests that the presence of SMC reduces the sealing properties of the endothelium, showing a more inflammatory phenotype while the distance between the two cell types reduced their interactions. These results demonstrate that EC-SMC interactions affect EC phenotype and change the EC response to WSS. Furthermore, the interactions formed between the EC and SMC demonstrate that the 1-side model can simulate better the arterioles, while the 2-side model provides better simulation of larger arteries.

## Introduction

The close proximity between the endothelial cells (EC) that compose the intima of blood vessels and the smooth muscle cells (SMC) composing the tunica media enables both these cells to interact with each other. Extensive research on the effects of physiological hemodynamic forces has found that WSS causes functional switching of the endothelial phenotype from a quiescent atheroprotective phenotype under physiological and elevated levels of WSS (10–20 dyne/cm^2^) to an atherogenic phenotype at low WSS (0–4 dyne/cm^2^) [1]–[3]. However, the impact of these SMC-EC interactions on the response of EC to WSS is still unclear and requires more research.

**Figure 1:**
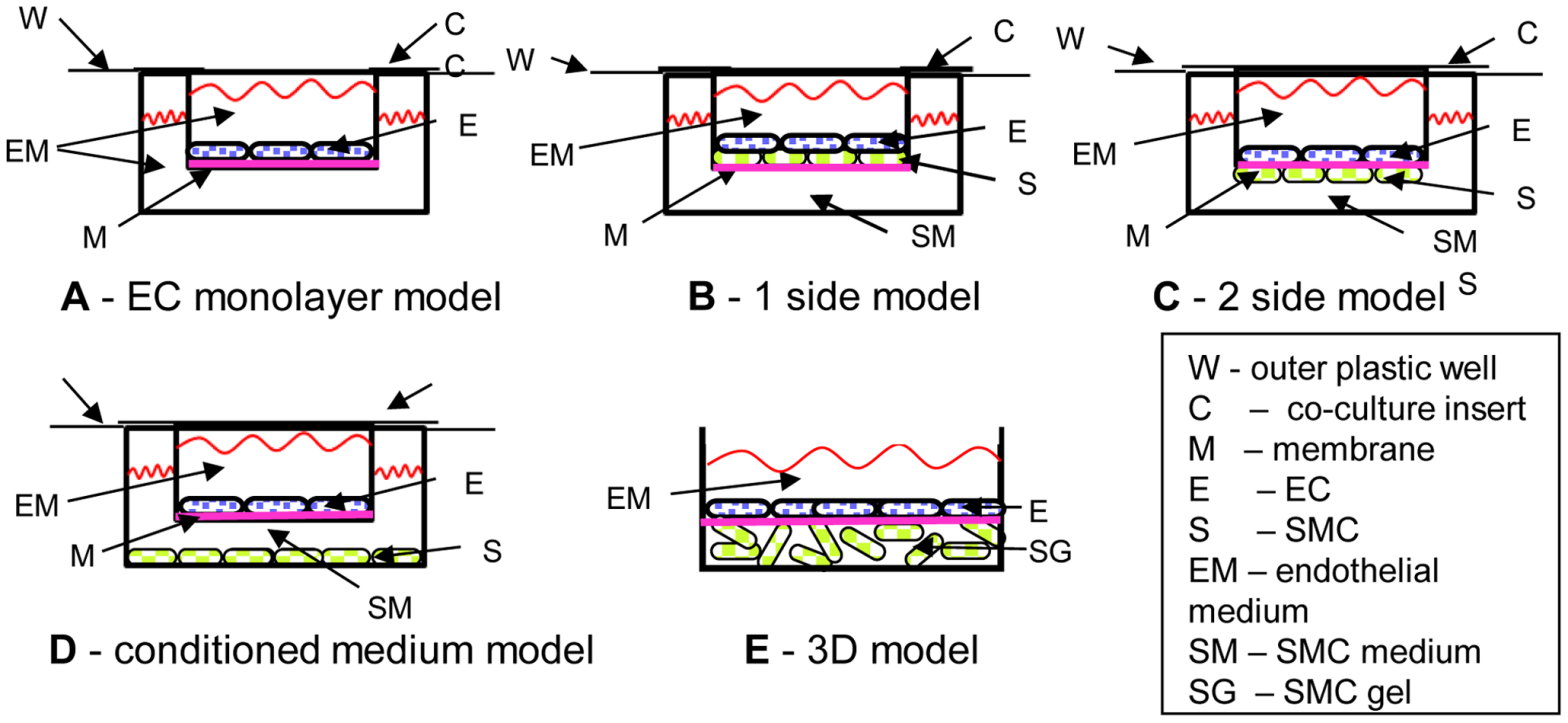
The major vascular wall models found in the literature. A - EC monolayer model, presented on a membrane but can be on the outer plastic as well. B – 1-side model, where EC are cultured on a confluent culture of SMC. C – 2-side model, where EC and SMC are cultured on opposite sides of a porous membrane. The cell can form connections and usually shares the same medium. D – Conditioned medium model, where the cells share the same medium, but no connections can be formed between the cells, and E – EC are cultured on a polymerized gel containing SMC, usually collagen gel.

**Figure 2:**
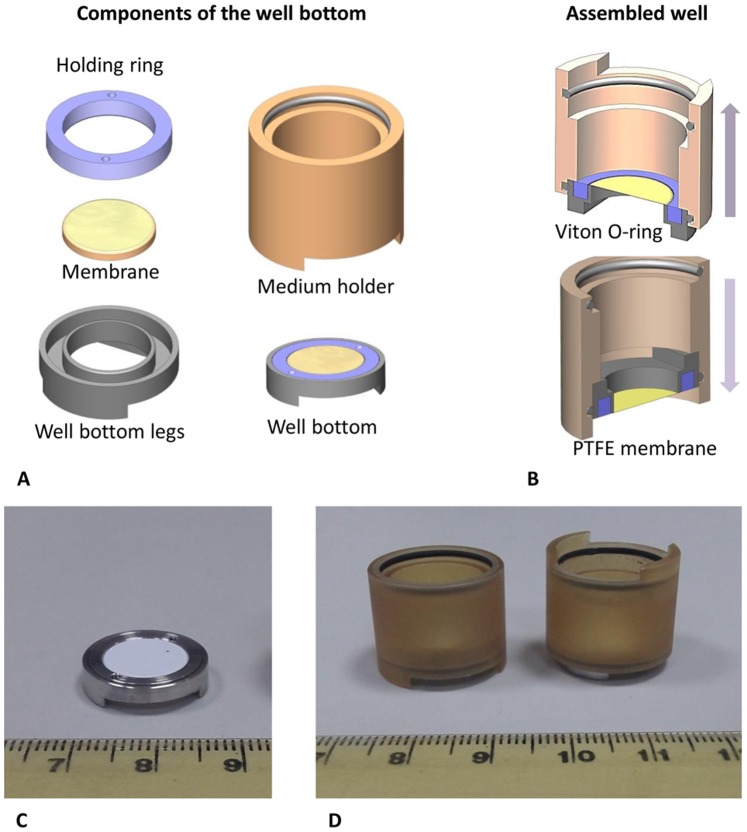
The custom made co-culture wells for the *in vitro* vascular wall models. A – Diagram of the parts of the well. B – Assembled well, top – upper side up, bottom – face down. C – Picture of the disassembled well bottom. D – Pictures of assembled well, right – upper side up, left – face down.

**Figure 3:**
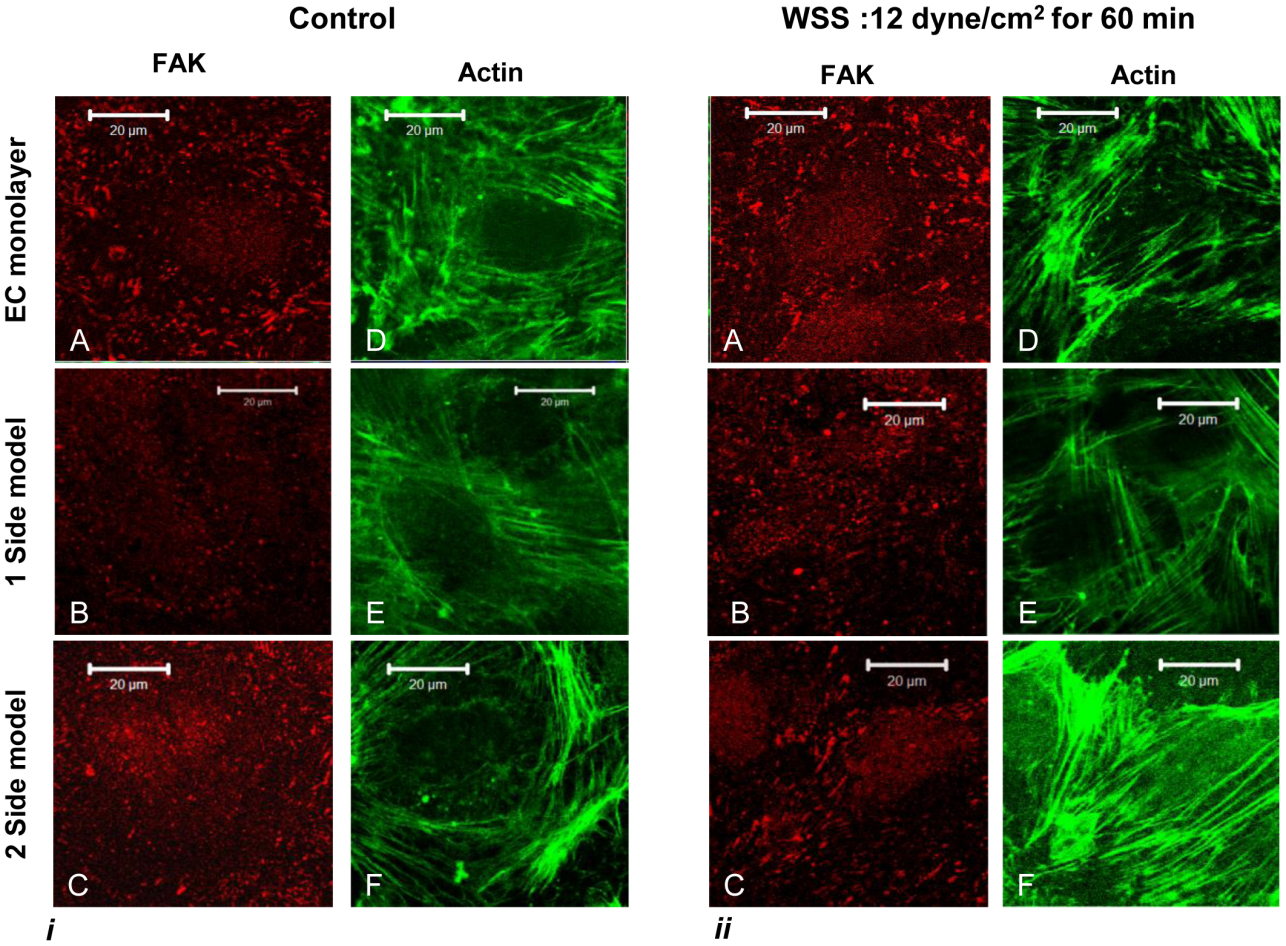
HUVEC stained for actin stress fibers (green) and FAK (red). i) no flow (control); ii) 60 min of WSS of 12 dyne/cm^2^. The FAK are located at the end of the actin fibers In the control samples the FAK form large plaques that are located closer to the cell contour, forming a wide band at the cell borders. The FAK in the 1-side model are smaller and limited compared to the other models, however they seem to re-appear following exposure to WSS. Subsequent to exposure to flow, the actin stress fibers are aligned in the flow direction with the FAK located closer to the cell borders.

Most research on the effects of WSS on EC biology has been done with EC alone, that only forms EC-EC interactions and without the interactions of EC with its neighboring SMC (EC-SMC interactions) [4]. To investigate the interactions between EC and SMC, several co-culture techniques and models have been introduced. The main four co-culture models are : (1) direct culture of EC on SMC (1-side model) as illustrated in Figure 1B; (2) a culture of SMC and EC on opposite sides of different membranes (2-side models) as illustrated in Figure 1C; (3) a culture of EC on collagen gels or other polymers containing SMC (3 dimensional (3D) model) as illustrated in Figure 1E; and (4) the use of conditioned media as illustrated in Figure 1D, which is a useful method for assessing EC–SMC interactions through secretory mechanisms [5]–[7].

The effect of WSS on various EC-EC interactions has mainly been studied on EC monolayers (Table 1). A short exposure to WSS was shown to induce a discontinuity in vascular endothelial (VE)-Cadherin staining at the border of the EC. This change was not accompanied by a change in VE-Cadherin expression [8]–[11]. Similarly, *in vivo*, VE-Cadherin expression was weak and discontinuous in areas of disturbed flow, but significantly higher and continuous where flow was laminar [8], [12]. In addition, connexin 43 (Cx43), which forms gap junctions between the EC (EEGJ), was found to be minimally expressed or absent when exposed to physiological WSS, whereas the gap junctions Cx37 and Cx40 were found to be up-regulated when exposed to physiological WSS. This altered pattern of Cx expression in EC was supported by *in vivo* findings [13]–[15]. Moreover, acute WSS increased EC permeability, which was mediated by nitric oxide (NO) [16], and occludin phosphorylation [17] on the other hand, chronic WSS decreased EC permeability, which was mediated by NO as well [18]. The Platelet Endothelial Cell Adhesion Molecule-1 (PECAM-1) was found to participate in a mechano-sensory complex that responded to WSS activation [19]; on the other hand, staining of PECAM-1 was reduced in the presence of SMC in a 1-side model [20].

**Table 1:** A summary of published endothelial-endothelial interactions examined in different vascular wall models.

Model Type	EC-EC Interactions	EC source	SMC source	References
**EC monolayer model** - (Fig. 1A)	Adherens junctions: VE-Cadherin and catenin biochemistryand effect of WSS	PTAEC, BAEC,HUVEC		[8], [11], [49]
	Gap junctions: connexins 37, 40, 43 endothelium derivedhyperpolarizing factor, permeability betweenthe cells (dye transfer) and effect of WSS	HAEC, bEnd.3,BAEC		[13], [42], [50]
	Endothelium permeability (tight Junctions: Zonnulaoccludens proteins) and the effect of WSS			Reviewed by[17], [18]
	PECAM-1 role as a WSS sensor and ininflammatory response.	BAEC		[19], reviewedby [51]
**1-Side** - (Fig. 1B)	LDL uptake/infiltration	BAEC	BASMC	[52], [53]
	PECAM-1	PCAEC	PCASMC	[20]
**2-Side** - (Fig. 1C)	LDL uptake and water infiltration	HUVEC	HUSMC	[52]
**3D-model** - (Fig. 1E)	Effect of WSS on myoendothelial Junctions(Cx37, 40 and 43) expression	HAEC	HASMC	[35]

Abbreviations: PTAEC-pig thoracic aorta EC, BAEC-bovine aortic EC, HUVEC-Human umbilical vein EC, HAEC-Human aortic EC, bEnd.3- PymT-transformed mouse endothelial cells clone bEnd.3, PCAEC-pig carotid artery EC, HUSMC-Human umbilical SMC, HASMC-Human aortic SMC, BASMC-Bovine aortic SMC, PCASMC-Pig carotid artery SMC.

The effect of WSS on the interactions between EC and SMC has been shown to reduce the inflammatory response induced by the proliferating SMC on the EC. This was evidenced by the reduced expression levels of adhesion molecules such as P-selectin and the vascular adhesion molecule (VCAM) [21], [22], a reduced fibrinolytic and coagulatory response [23], reduced cell migration and proliferation rate [4], [24], as well as the EC and SMC gene profiles [25], [26] following exposure to WSS. Table 2 summarizes these main EC-SMC interactions. In addition, Wallace et al. found that EC attached to SMC using integrin α5β1 and lacked focal adhesions in the 1-side model [27], but it was still attached firmly to SMC with very limited cell loss following exposure to high WSS [20], [28]. By contrast, in a 2-side model, the presence of SMC increased the number of adherent ECs, as well as the total focal adhesion area in EC [29].

**Table 2:** A summary of published endothelial-smooth muscle cell interactions examined in different vascular wall models.

Model Type	EC-SMC Interactions	EC source	SMC source	References
**1-Side** - (Fig. 1B)	EC adhesion to SMC	PCAEC, HAEC,HUVEC	PCASMC, HASMC	[20], [27], [28], [54]
	WSS effect on LDL uptake	BAEC	BASMC	[55]
	EC elastic modulus	HCAEC	HASMC	[56]
	WSS effect on coagulation and fibrinolytic factors	HSVEC	HSVSMC	[23]
	WSS effect on SMC and EC gene profile	HSVEC	HSVSMC	[25], [26]
**2-Side** - (Fig. 1C)	Myoendothelial Junctions (Cx37, 40 and 43) organizationand regulation in Static conditions	MAEC, RPAEC,HUVEC	MASMC, RPASMC, HUSMC	[30]–[34]
	WSS effect on EC and SMC migration and proliferation	HUVEC	HUSMC	[4], [24], [57], [58]
	SMC differentiation	BAEC, HAEC	BASMC, HASMC	[6], [59], [60]
	Effect of WSS on SMC phenotype	HUVEC	HUSMC	[61]
	SMC and EC adhesion to matrix	HUVEC	HUSMC	[29], [62]
	Effect of WSS on EC gene and proteins expression ofadhesion molecules	HUVEC	HUSMC	[22], [63], [64]
	Direct contacts through Jagged 1 protein importancein SMC differentiation	HCAEC	HCASMC	[65]
	Effect of WSS on SMC gene expression	HUVEC	HUSMC	[26]
**Conditioned Medium –**(Fig. 1D)	WSS on EC change SMC proliferation and migration	HUVEC	HUSMC	[57]
	SMC differentiation	BAEC, HUVEC	BASMC, HUSMC	[6], [66], [67]
**3D-model** - (Fig. 1E)	Effect of WSS on myoendothelial Junctions(Cx37, 40 and 43) expression	HAEC	HASMC	[35]
	Effect of WSS on EC expression of adhesion molecules	HUVEC	HUSMC	[21]
	Effect of WSS on EC proliferation and migration	HCAEC	HASMC	[68]
	WSS effect on SMC phenotype	BPAEC	BPASMC	[69], [70]

Abbreviations: PTAEC-pig thoracic aorta EC, BAEC-bovine aortic EC, HUVEC-Human umbilical vein EC, HAEC-Human aortic EC, PCAEC-pig carotid artery EC, HSVEC-Human saphenous vein EC, MAEC-Mouse aorta EC, RPAEC-Rat pulmonary artery EC, HCAEC-Human carotid artery EC, BPAEC-Bovine pulmonary artery EC, HUSMC-Human umbilical SMC, HASMC-Human aortic SMC, BASMC-Bovine aortic SMC, PCASMC-Pig carotid artery SMC, HSVSMC- Human saphenous vein EC, MASMC- Mouse aorta SMC, RPASMC- Rat pulmonary artery SMC, HCSMC- Human carotid artery SMC, BPAEC- Bovine pulmonary artery SMC.

The direct communication of EC-SMC through myoendothelial gap junctions (MEGJ) has mainly been studied without exposure to WSS (Table 2). Isakson et al. reported the presence of Cx37, Cx40 and Cx43 between the 2 cell types using the 2-side model. In this model, the EC and SMC form direct connections via cellular projections through the membrane pores [30]. Further studies have explored different biochemical activations and regulations of the MEGJ in this model without the effect of WSS [31]–[34]. Johnson and Nerem used a 3D model and found an up-regulation in Cx37, Cx40 and Cx43 expression in the presence of SMC, and that Cx37 expression was even higher following exposure to WSS, whereas Cx43 was down-regulated by the flow. The increased expression of connexins of EC in the presence of SMC was probably due to the role of the connexins in the MEGJ [35]. However, the presence of the MEGJ has not been studied in the 1-side model and it is not clear whether the interactions between the EC and SMC in the different models are similar; moreover, the effect of WSS exposure on the EC-SMC connections in the 1-side and 2-side model awaits investigation.

Most studies on WSS effects on the EC-SMC interaction have focused on the inflammatory response of both EC and SMC. However, those studies have not considered that in vivo, the EC-SMC interactions may vary between different types of blood vessels, affecting the response of EC to WSS. Based on the understanding of these interactions in vivo, we hypothesized that for the studies of the effects of mechanical forces on the endothelium the 1-side model can simulate better the arterioles, while the 2-side model provides better simulation of larger arteries. Thus, the main goal of this work was to study the ways in which the interactions between EC and their neighboring cells (i.e. EC-SMC interactions) affect the response of EC-EC interactions (i.e. VE-Cadherin) and EC-SMC (i.e. MEGJ) to WSS stimulation. To support our hypothesis we compared the response of EC to WSS using 3 different models: (1) EC monolayer, (2) 2-side model, and (3) 1-side model. The models were exposed to a low WSS of 4 dyne/cm^2^, a physiological WSS of 12 dyne/cm^2^ and a control level (0 dyne/cm^2^). We analyzed the morphology of the models and the expression levels of proteins responsible for the cell interactions with their neighboring cells.

## Methods

### Custom-designed Wells for the in-vitro Cell Culture Models

The cell cultures for the *in vitro* models of the vascular wall in this work were grown in modified wells that were previously developed in our laboratory [36]. Briefly, these wells are constructed from a well bottom and a cylinder. The well bottom holds a stretched polytetrafluoroethylene (PTFE) membrane that can be used as a substrate for the cell culture and the cylinder is assembled in such a way as to hold the medium above the membrane. The cylinder is constructed so that the well bottoms can be assembled on both sides (Fig. 2). This makes it possible to culture cells on both sides of the membrane. Once the co-cultured layers become confluent and ready for flow experiments the cylindrical medium holder can be disassembled from the well bottom (Fig. 2) and installed in the flow chamber for flow experiments. Upon completion of the experiments, the medium holder can be re-assembled on the well bottom for either biological treatment of the cells or for further culture. The PTFE membrane (Millipore) was coated using 10% fibronectin (Biological Industries, Beit Haemek, Israel) solution prior to cell culturing.

### Human Umbilical Vein Endothelial Cells and Human Umbilical Artery Smooth Muscle Cells

Human umbilical vein endothelial cells (HUVEC) (PromoCell) were cultured in endothelial cell media (PromoCell) supplemented with 100 U/ml Penicillin, 0.1 mg/ml Streptomycin and 12.5 U/ml Nystatin (Biological Industries, Beit Haemek, Israel).

Human umbilical aortic smooth muscle cells (SMC) (Lonza) were cultured in MCDB-131 Medium supplemented with 5% Fetal Bovine Serum, 2 mM L-Glutamine, 100 U/ml Penicillin, 0.1 mg/ml Streptomycin and 12.5 U/ml Nystatin (Biological Industries, Beit Haemek, Israel), 2 ng/ml Insulin (Sigma Aldrich), 0.5 ng/ml Epithelial Growth Factor (Sigma Aldrich), 3 ng/ml Basic-Fibroblasts Growth Factor (bFGF) (PeproTech Inc., USA) and 1 µg/ml Hydrocortisone (Sigma Aldrich). Both cell types as well as the co-culture were cultured at 37°C and 5% CO_2_ in a humidifier incubator. In both cell types, only passages 4–8 were used for this study.

### The *in-vitro* Vascular Wall Models

#### The 2-side co-culture model

The PTFE membrane was installed in the custom-designed well facing downwards and was placed into a plastic tissue culture dish containing the SMC growth medium (Fig. 2B bottom and Fig. 2D left). About 7.5×10^4^ SMCs were seeded on the PTFE membrane on the lower side of the well. After 24 hours, the membrane was washed to remove excess non-adherent SMC and the well was re-assembled so that the SMC cells would be facing downwards and completely submerged in the HUVEC growth medium (Fig. 2B top and Fig. 2D right). Then, 10^5^ HUVEC were seeded on the membrane side facing upward. After 24 hours, both layers of EC and SMC became confluent and ready for the WSS experiments.

#### The 1-side co-culture model

The PTFE membrane was installed in the custom-designed well with the upper face upwards and was placed into a plastic tissue culture dish containing the SMC growth medium. About 7.5×10^4^ SMC were seeded on the PTFE membrane on the upper side (Fig. 2B top and Fig. 2D right). After 24 hours, the membrane was washed to remove excess non-adherent SMC and the medium was replaced by HUVEC growth medium. Then, 10^5^ HUVEC were seeded on top of the SMC layer (upper side). After 24 hours both layers of EC and SMC became confluent and ready for WSS experiments.

#### The endothelial monolayer model

The PTFE membrane was installed in the custom-designed well with the upper face upwards and was placed into a plastic tissue culture dish containing the HUVEC growth medium (Fig. 2B top and Fig. 2D right). About 10^5^ HUVEC were seeded on the PTFE membrane (upper side). At this step we ensured that the medium completely covered the underside of the membrane with the cell culture and without any gas bubbles. After 24 hours the HUVEC layer became confluent and ready for WSS experiments.

Once confluent layers were obtained (approximately 16 hours after the EC were seeded), the custom-designed wells were disassembled and the well bottom with the *in vitro* vascular wall model was assembled in the flow chamber, so that the HUVEC side would be exposed to physiological flows while the other side (with no cells or with SMC) would stay in static conditions. Prior to conducting the experiments, all models were inspected under phase contrast microscopy to verify that the cells had reached at least 90% confluency.

### Flow System for Wall Shear Stress Experiments

A custom designed parallel plate flow chamber with a rectangular cross section was built to apply uniform laminar WSS on 3 samples of the tissue-engineered vascular wall models. Since the height (*h* = 1 mm) of the flow chamber is much smaller than either its length (l = 100 mm) or its width (*b* = 35 mm) the fluid mechanics of the channel can be described as a parallel plate flow chamber. Thus, the WSS (τ_ω_) is given by:
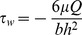
(1)where Q is the flow rate and μ = 0.007 dyne*s/cm^2^ is the fluid viscosity for the medium at 37°C.

In addition, a computational fluid dynamics (CFD) model was developed to validate the uniform distribution of the WSS in the flow chamber, using the computational fluid dynamics package of FLUENT (Fluent Inc., Lebanon, NH). The flow profiles at different locations were computed and compared for a uniform inlet velocity of *U_x_ = *0.3125 m/s, which corresponds to a flow rate of *Q_in_* = 1.2 L/min, for which Re = 1628 and the analytically calculated WSS equaled 24 dyne/cm^2^. The WSS distributions on the bottom of the flow chamber were computed for three axial locations that corresponded to the center of each of the three well bottoms and showed a uniform WSS field of 24 dyne/cm^2^ over the entire surface of all three well bottoms.

For the WSS experiments, the well bottoms were inserted into the bottom part of the flow chamber so that the PTFE membrane was in the same plane as the chamber bottom, allowing for laminar flow on the HUVEC side of the vascular wall models. The flow chamber was then connected with tubes to a peristaltic pump and a medium reservoir. The flow chamber and the medium reservoir were maintained at 37°C and 5% CO_2_ in a humidifier incubator during exposure to WSS of the endothelial side of the models. The flow chamber allows for up to 3 wells in each flow experiment. All experiments were performed using a flow rate of 600 ml/min resulting in a WSS of 12 dyne/cm^2^ for physiological WSS, a flow rate of 200 ml/min resulting in a WSS of 4 dyne/cm^2^ for low WSS and, static conditions (i.e. 0 dyne/cm^2^) as the control. The WSS experiments were conducted for 30 min in the case of the connexins protein quantification, and for 30 and 60 min for visualization of cell morphology, VE-Cadherin and FAK. All experiments were performed using all three models of the vascular wall as described above and all the experiments were performed at least three independent times with three wells in each test.

### Cell Morphology (Immunofluorescence Stains)

The vascular wall models were exposed for 30 and 60 min to 0, 4 and 12 dyne/cm^2^ and then fixed in a 4% paraformaldehyde solution for 10 min. This was followed by blocking, using a phosphate buffered solution (PBS) containing 10% normal goat serum and 0.1% Triton X-100 solution for 30 min followed by incubation overnight at 4°C with the following primary antibodies: a mouse anti VE-Cadherin (Santa Cruz Biotechnology) and a rabbit anti FAK (Abcam). This was followed by an incubation of 30 min with a secondary antibody: alexa fluor 647 conjugated goat anti rabbit, alexa fluor 488 conjugated goat anti mouse (Jackson ImmunoResearch) and TRITC conjugated phalloidine (Sigma Aldrich). Between each step the samples were rinsed 3 times with PBS. The samples were then mounted between 2 large microscope glass slides using a DAPI mounting gel (Santa Cruz Biotechnology) in order to visualize the cell nucleus. The slides were stored in a covered box at 4°C until microscopy examination. The cells were examined under a Zeiss LSM510 confocal microscope. Analysis and quantification of the images was done using the LSM image browser software (Zeiss) and the provided measuring tools.

### Protein Quantification (Flow Cytometry)

The vascular wall models were exposed for 30 min to 0, 4 and 12 dyne/cm^2^ and then the cells were trypsinized and centrifuged for 5 min at 7 g to separate the cells from the trypsin solution. The cell suspensions of both HUVEC and SMC were then fixed and permeabilized using ice cold methanol for 10 min at −20°C. The samples were incubated first with the appropriate primary antibody, followed by incubation with the appropriate secondary antibodies for 30 min each at room temperature. Following each step, 1 ml of PBS was added to the cell suspension and the resulting solution was then centrifuged for 5 min at 7 g to separate the cells from the solution. The solution was gently poured out of the test tube, leaving the cell pellet at the bottom of the test tube. The cells were mixed to form a homogenous cell suspension using a vortex after the next solution was added. In order to differentiate the different cells, each sample was labeled for VE-Cadherin (which is positive for HUVEC and negative for SMC) using a mouse anti VE-Cadherin antibody (Santa Cruz) and for one of the following primary antibodies: rabbit anti Cx37 alexa fluor 647 conjugated (BIOS), rabbit anti Cx43 alexa fluor 488 conjugated (BIOS) followed by the secondary antibodies: alexa fluor 647 conjugated goat anti rabbit and alexa fluor 488 or alexa fluor 647 conjugated goat anti mouse. Positive control samples were labeled with primary and secondary antibodies and negative control samples were labeled with secondary antibody alone. Samples were analyzed using a BD LSR digital flow cytometer. The Flowing software (Turko University, Finland) was used for data analysis.

### Statistical Analysis

The results were evaluated using a 2-way analysis of variance (SPSS, IBM v19). A *P* value of less than 0.05 was considered statistically significant. Post-hoc Tukey-Kramer tests supplied by the SPSS software were used to perform pair wise multiple comparisons between the data obtained from the different WSS applied on the different models.

## Results

We investigated the attachment of EC to the matrix as well as the interactions of EC-EC and EC-SMC in response to WSS stimulation. For this purpose we compared the morphology and amount of proteins immediately following short term exposure to different WSS in three different models of the vascular wall made up of EC monolayer and two co-cultures of EC and SMC in which EC were cultured directly over a confluent layer of SMC or on the opposite side of a membrane with a culture of SMC.

### Endothelial-matrix Interactions

The focal adhesion kinase (FAK), which is an important protein in the focal adhesion complex that attaches cells to the matrix, was used to evaluate the EC-matrix interaction. Stains of HUVEC actin stress fibers and FAK, following the exposure to a WSS of 12 dyne/cm^2^ for 0 and 60 min are shown in Figure 3. The FAK stains shows that the focal adhesions are located at the end of the stress fibers, forming a wide band at the cell borders as can be seen in Figure 3i-A to C, with fewer and smaller apparent plaques in the 1-side model in the control compared to the amount of plaques in the EC monolayer and the 2-side models (Fig. 3i - B). Following exposure to a WSS of 12 dyne/cm^2^, the HUVEC became a little more elongated and the stress fibers aligned with the flow direction in all three models (Fig. 3ii). The stress fibers aligned with the cell direction and formed longer and less peripheral fibers (Fig. 3ii A to C). Following exposure to WSS, in the 1-side model, the FAK stain started to resemble the other models (Fig. 3ii - B).

**Figure 4:**
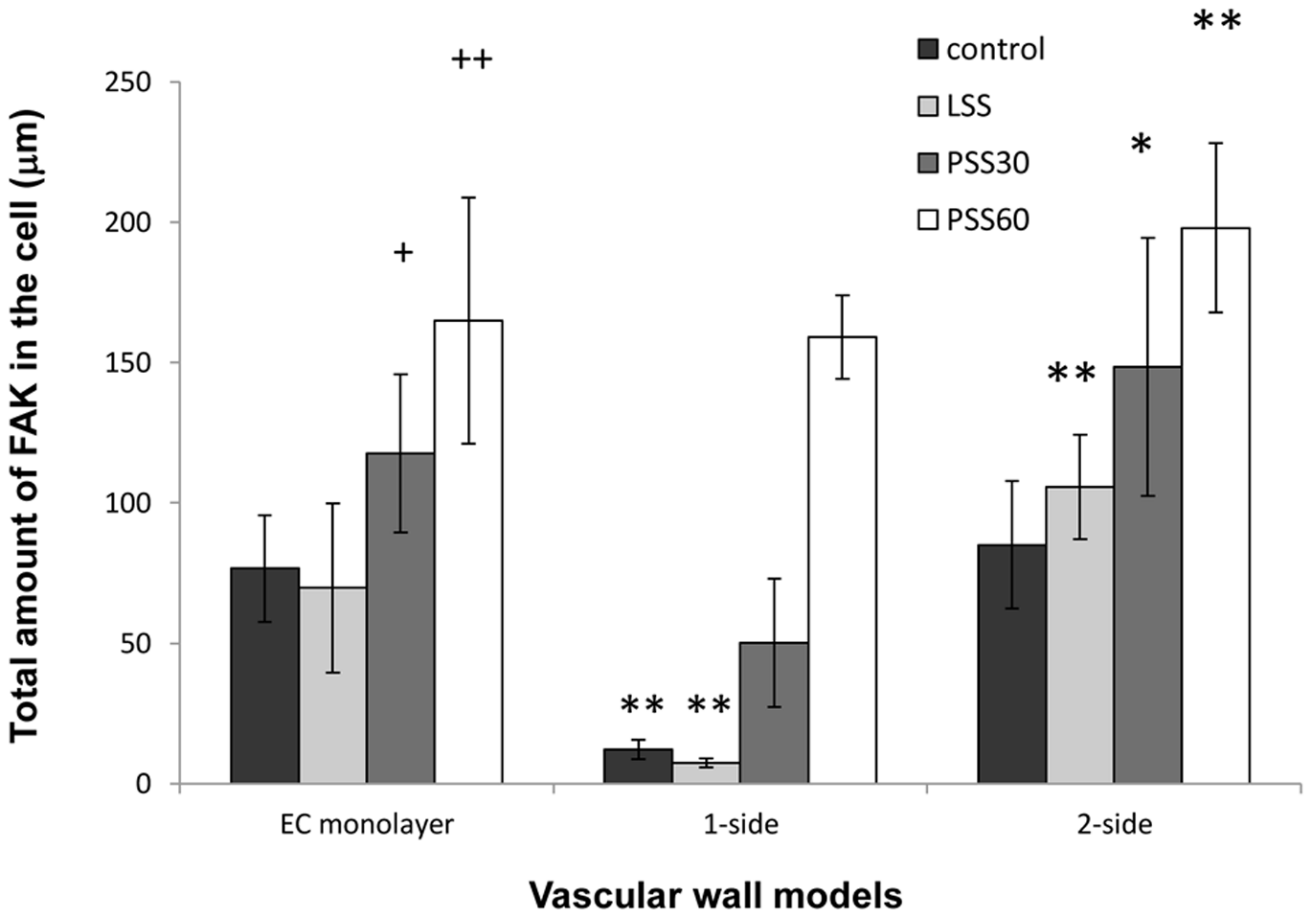
The total amount of FAK per cell in the three vascular wall models. Total amount of FAK per cell distribution in the different models in static conditions and following exposure to WSS of 4 dyne/cm^2^ for 30 min (LSS) and to WSS of 12 dyne/cm^2^ for 30 (PSS30) and 60 min (PSS60). The average amount of FAK per cells was calculated by multiplying the average FAK plaque length and average amount of plaques per cell, taken from measured data produced from the FAK images. The amount of FAK increased following exposure to 12 dyne/cm^2^ in a time dependent manner, but was not affected by exposure to 4 dyne/cm^2^ in any of the three models. The amount of FAK in the 1-side model was the lowest in the control as well as following exposure to 30 min of 4 and 12 dyne/cm^2^. The amount of FAK in the 2-side model was the highest in all WSS exposure modes. (+ - P<0.05 compared to the same model without WSS. ++ - P<0.001 compared to the same model without WSS. * - P<0.05 compared to the EC monolayer model following the same exposure to WSS. ** - P<0.001 compared to the EC monolayer model following the same exposure to WSS).

The length and amount of FAK plaques were measured from the images of the vascular wall models. For each field the average plaque length and amount of plaques per cell were calculated and used to estimate the equivalent average total amount of FAK per cell (Fig. 4). The results showed that in all three models, the exposure to a WSS of 12 dyne/cm^2^ led to an increase in the amount of FAK in a time dependent manner. In static conditions and following exposure to 30 min of 4 dyne/cm^2^ (LSS) the amount of FAK in the 1-side model was very low (significantly different from the other models). Following an exposure of 30 min to 12 dyne/cm^2^ (PSS30) the amount of FAK in this model increased (significantly); however it was still lower than in the other two models. Following an exposure of 60 min to 12 dyne/cm^2^ (PSS60), however, the amount of FAK was similar to that of the EC monolayer model. The amount of FAK in the 2-side model was a little higher in all modes of WSS exposure (Fig. 4).

### Endothelial-endothelial Interactions

The VE-Cadherin, which is the main adherens junction of the endothelium, was used to evaluate the EC-EC interaction, since these junctions are the major contributors to the cell–cell adhesion force. In addition, the gap junction proteins Cx37 and Cx43 were used to evaluate the EC-EC communication.

**Figure 5:**
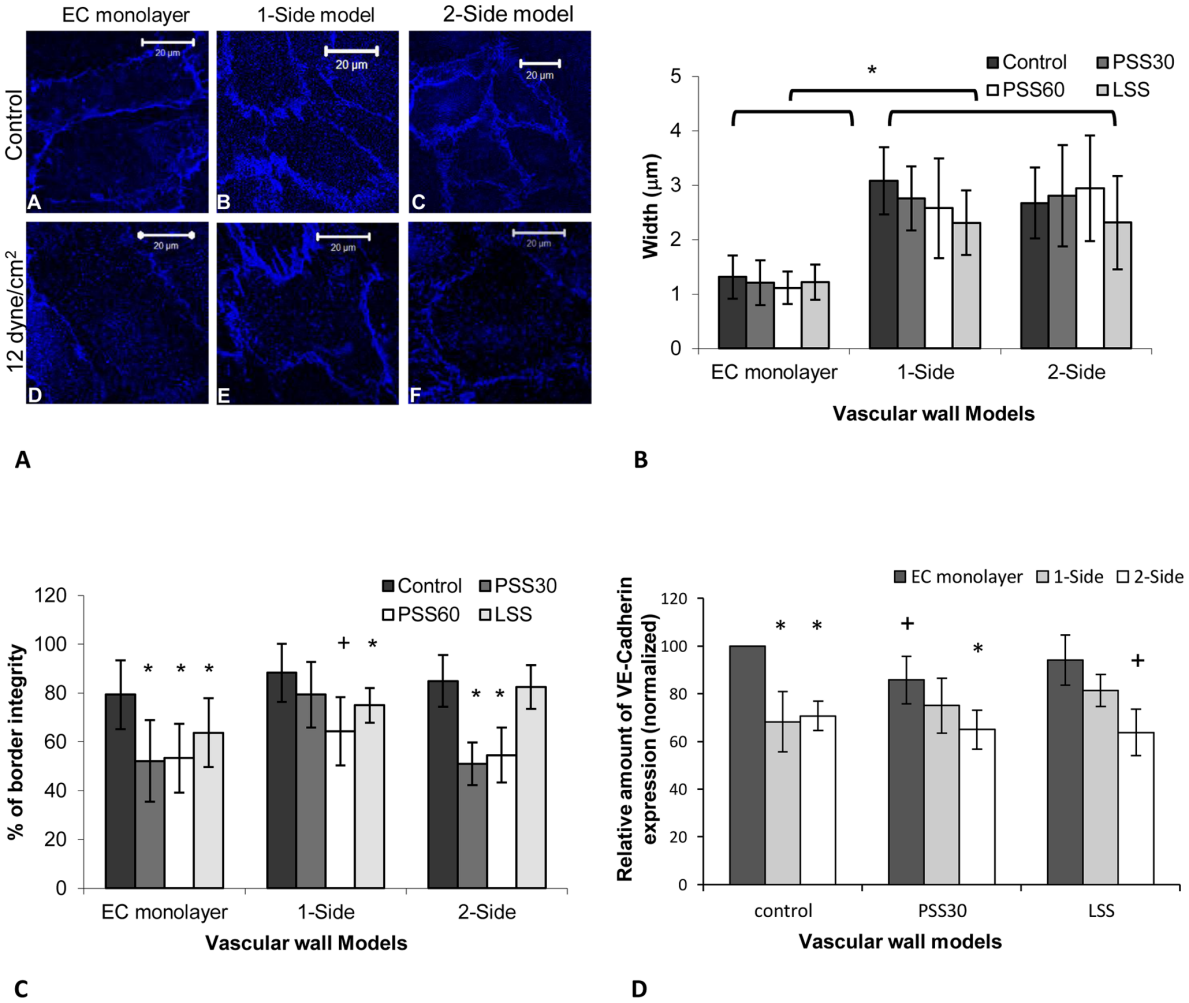
VE-Cadherin analysis. A **-** The VE-Cadherin marks the border of the cells, with a wider mark of the cell borders in the 1 and 2-side models (B and C). Following exposure to WSS there was a dissociation of the VE-Cadherin stain (D–F). B - The stain thickness distribution, as measured from the images of the VE-Cadherin, in the different models with and without exposure to WSS. The thickness distribution in the EC monolayer was around 1 µm, whereas in the co-culture models it was roughly 2 to 3 µm. The difference across models was significance. C **-** The continuity in the vascular wall models, as estimated from the VE-Cadherin images, following exposure to a WSS of 12 dyne/cm^2^ for 30 and 60 min and a WSS of 4 dyne/cm^2^ for 30 min and without WSS exposure. VE-Cadherin continuity was high in static conditions and declined when the cells were exposed to a WSS of 12 dyne/cm^2^, with a significant effect in the 1-side model alone following 60 min of exposure. Following exposure to a WSS of 4 dyne/cm^2^ this continuity only decline significantly in the EC monolayer model. D - VE-Cadherin expression in the endothelial cells of the different models following exposure to 30 min of a WSS of 4 and 12 dyne/cm^2^ and without exposure to WSS. The change in VE-Cadherin expression was significant when comparing the EC monolayer model to the two co-culture models; however the change in the amount of VE-Cadherin did not vary significantly in each model following exposure to WSS. The presented data is normalized to the EC-monolayer result in static conditions (control) sample. LSS - WSS of 4 dyne/cm^2^ for 30 min; PSS30 - WSS of 12 dyne/cm^2^ for 30 and PSS60 - of 12 dyne/cm^2^ for 60 min. (+ - P<0.05 compared to the same model without WSS. * - P<0.05 compared to the EC monolayer model following the same exposure to WSS).

Figure 5A shows the endothelial plane of the models when stained for VE-Cadherin. The VE-Cadherin marks the line where HUVEC are connected to one another, demonstrating the cell borders, and showing a confluent, mature endothelium in all three models. The width of the VE-Cadherin mark in the 1-side and 2-side models was much thicker than in the EC monolayer model. The VE-Cadherin in these two models seemed to be either double or had protrusions between two adjacent cells. Measurement of the stain thickness showed that the distribution of the thickness values of the stain in the EC monolayer model was around 1 µm whereas that of the 1-side and 2-side models was around 2 to 3 µm, hence showing a significance increase in the thickness of the stain that marks the connection between the cells (Fig. 5B). This thickness remained visible following exposure to WSS; however a slight (non-significant) decrease in this value emerged in the 1-side model.

Following exposure to a WSS of 12 dyne/cm^2^ for 60 min, the VE-Cadherin stain seemed less connected (Fig. 5A–D, E and F), and took on a fenestrated appearance. This discontinuity in the 1-side model was not as large as in the other models (Fig. 5A–E). Quantification of the integrity of the stain was done using a scale where 100% symbolizes a fully continuous line and a 0% symbolizes no line. The results showed that under static conditions, in all three models the integrity of the stain was around 80%; following exposure to a WSS of 12 dyne/cm^2^ the EC monolayer and 2-side monolayer had a 50% score for both periods of 30 min and 60 min of WSS exposure. The 1-side model integrity varied significantly only following 60 min of exposure to a WSS of 12 dyne/cm^2^ to a value of 65%. Following exposure to a WSS of 4 dyne/cm^2^ for 30 min, only the EC monolayer showed a significance difference in the integrity of the stain to about 65% (Fig. 5C).

The quantification of VE-Cadherin expression using FACS indicated a significantly higher amount of protein in the EC monolayer than in the two co-culture models. This difference remained significant following exposure to WSS in the 2-side model, where the exposure to WSS did not change the amount of VE-Cadherin expression significantly. A slight increase in the amount of VE-Cadherin expression in the 1-side model (not significant) emerged following exposure to a WSS of 12 dyne/cm^2^ with another increase following a WSS of 4 dyne/cm^2^ (not significant). Following exposure to a WSS of 12 dyne/cm^2^ a small reduction in the expression of VE-Cadherin was found in the EC monolayer model with a non-significant reduction in the case of 4 dyne/cm^2^ (Fig. 5D).

**Figure 6:**
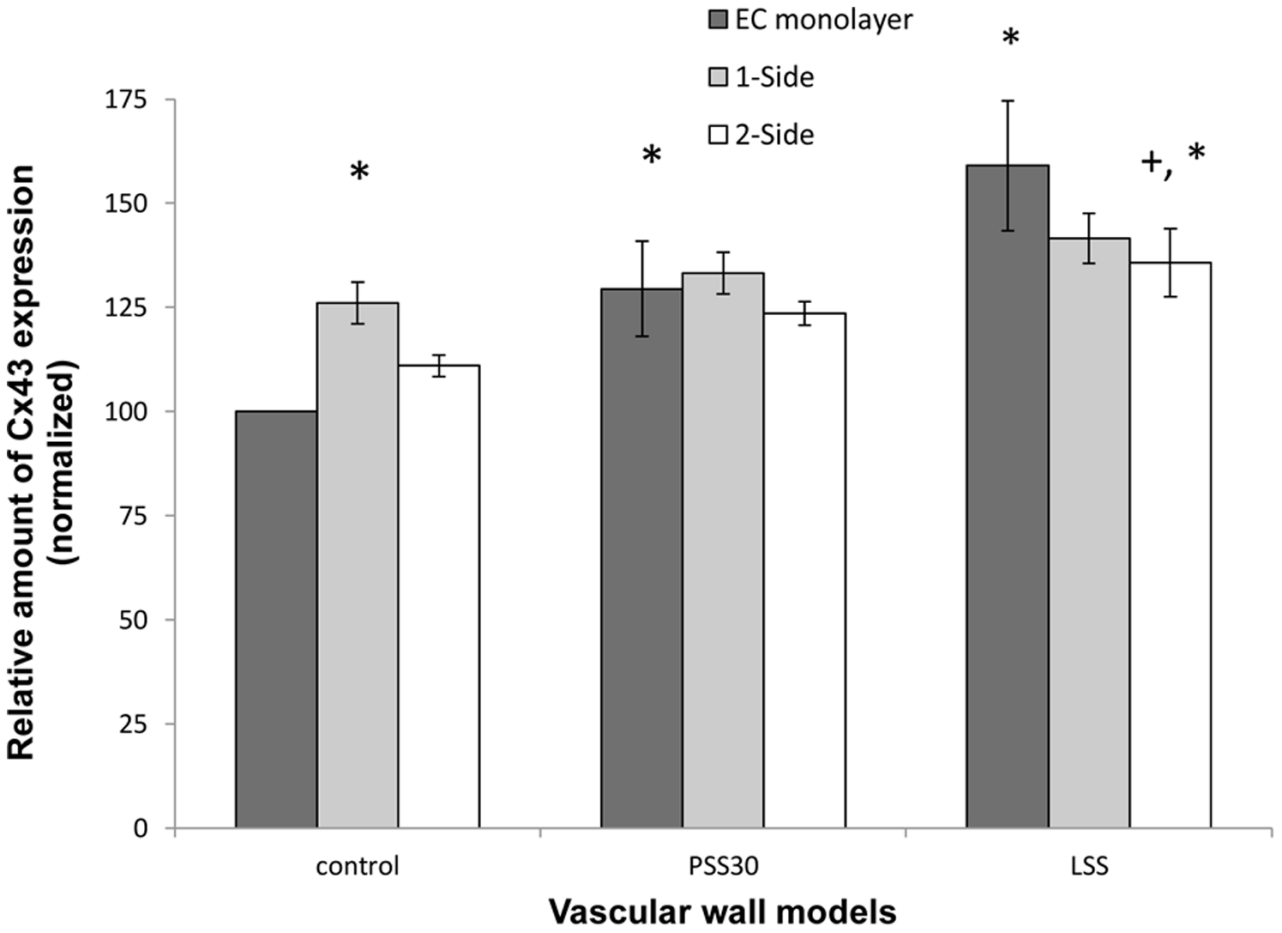
Cx43 expression in the endothelial cells of the different models. When the models were not exposed to WSS, the amount of Cx43 was higher in the co-culture models. This change was significant (P<0.01) in the 1-side model, but not in the 2-side model. Following exposure to 30 min of WSS of 12 dyne/cm^2^ the amount of Cx43 increased significantly, but only in the EC monolayer model, where it reached the same level as in the 2 other models. This increase was even higher following exposure to 4 dyne/cm^2^. Following exposure to 4 dyne/cm^2^ the 2-side model also increased significantly compared to the control. The presented data is normalized to the EC-monolayer result in static conditions (control) sample. LSS - WSS of 4 dyne/cm^2^ for 30 min; PSS30 - WSS of 12 dyne/cm^2^ for 30 and PSS60 - of 12 dyne/cm^2^ for 60 min. (* - P<0.05 compared to the control group. + - P<0.05 compared to the EC monolayer model in the same model).

**Figure 7:**
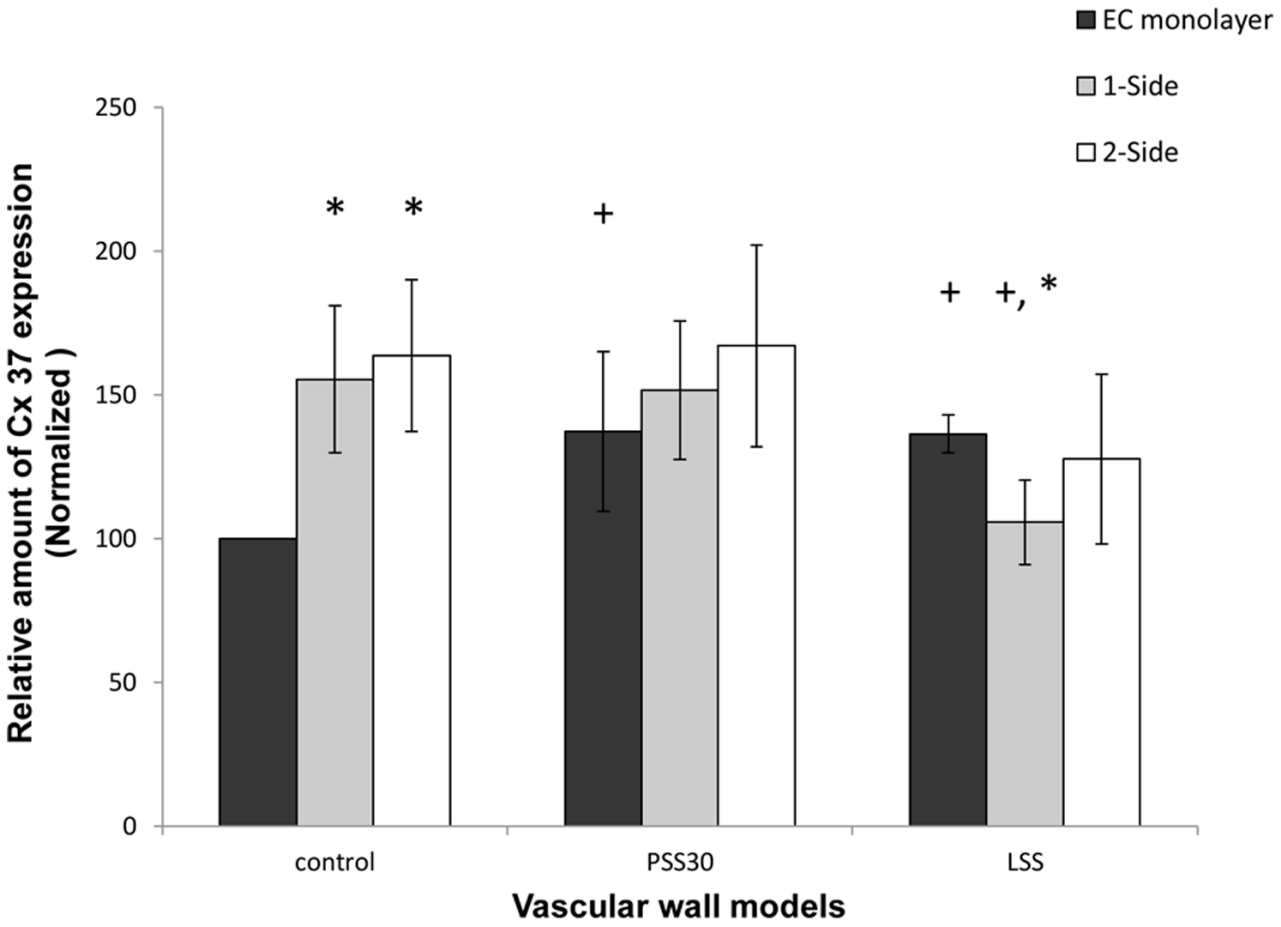
Cx37 expression in the endothelial cells of the different models. When the models were not exposed to WSS the amount of Cx37 was significantly (P<0.01) higher in the co-culture models compared to the EC monolayer model. Following exposure to 30 min of 12 dyne/cm^2^ and to 4 dyne/cm^2^the amount of Cx37 increased significantly in the EC monolayer model. In the 1-side and 2-side models there was no change following exposure to 12 dyne/cm^2^; however, following exposure to 4 dyne/cm^2^ the amount of Cx37 decreased significantly in the 1-side model, but non-significantly in the 2-side model. The presented data is normalized to the EC-monolayer result in static conditions (control) sample. LSS - WSS of 4 dyne/cm^2^ for 30 min; PSS30 - WSS of 12 dyne/cm^2^ for 30 and PSS60 - of 12 dyne/cm^2^ for 60 min. (* - P<0.05 within the control group. + P<0.05 compared to the control sample in the same model).

EC-EC communications increased following exposure to flow, as can be seen by the increase in the amount of Cx43 and Cx37, which formed EEGJ in the EC monolayer model following exposure to a WSS of 12 dyne/cm^2^ and 4 dyne/cm^2^ (Fig. 6 and 7), with a greater increase following exposure to 4 dyne/cm^2^ in the case of Cx43 (Fig. 6).

### Endothelial-smooth Muscle Cell Interactions

The myoendothelial gap junction proteins Cx37 and Cx43 form direct channels between EC and SMC. Thus, the amount of Cx37 and 43 was used to evaluate the EC-SMC direct interactions in the co-culture models. In order to calculate a statistic response of the cells, each sample was normalized to the EC-monolayer in static conditions (control) sample. The normalized results of the three repeats of the same test (non dependant repeats) were than averaged and presented (Fig. 6 and 7).

In static conditions, the amount of Cx43 showed a slight increase in the 2-side model compared to the EC monolayer and a significant increase in the 1-side model (Fig. 6), and there was a significant increase in the amount of Cx37 in both the 2-side and 1-side models, although there was no significant difference between the 2-side and 1-side models.

Following exposure to a WSS of 12 dyne/cm^2^ the amount of both Cx43 and Cx37 did not change significantly in either the 1-side and 2-side models (Fig. 6 and 7). However, following exposure to 4 dyne/cm^2^ the amount of Cx43 increased (significantly only in the 2-side model) (Fig. 6) and decreased (significantly only in the 1-side model) (Fig. 7).

## Discussion

In this study we have studied how WSS affect the interactions of the endothelium with its surrounding cells, which constructs the vascular wall. This was done using two different co-culture models of the vascular wall as well as a control model, which lacks the interaction with SMC. The results show that EC attaches to the SMC using a different FA complex, and not using FAK. Following exposure to a WSS of 12 dyne/cm^2^ the FAK became longer and increased in its amount in a time dependent manner. In the 1-side model, this exposure caused the appearance of the FAK until it resembled the EC monolayer model. In addition, the EC-EC interactions were reduced by the presence of SMC, as demonstrated by the amount of VE-Cadherin and stain. However, when exposed to WSS, the interactions with the SMC reduced the discontinuity in EC-EC interactions seen in the VE-Cadherin stain. In the presence of SMC the EC formed direct EC-SMC interactions using both Cx37 and Cx43 to form MEGJ. When exposed to a WSS of 12 dyne/cm^2^ both Cx43 and Cx37 expression were elevated in the EC monolayer, suggesting higher connectivity of EEGJ, however without a significant effect in the co-culture models where it formed EC-SMC interactions. When exposed to a WSS of 4 dyne/cm^2^ the amount of Cx43 was elevated in all 3 models, with the highest in the EC monolayer and lowest in the 1-side model, suggesting that the interactions with SMC reduce the effect of WSS. This effect was more pronounced when there was no distance between the two cell types (caused by the membrane between the cells in the 2-side model). In the case of Cx37, a WSS of 4 dyne/cm^2^ increased Cx37 expression compared to the control in the EC monolayer (demonstrating higher EEGJ interactions); however, in the co-culture models (where it formed EC-SMC interactions), the expression of Cx37 was reduced, with a greater difference in the 1-side model, showing that again, a distance between the 2 type of cells due to the presence of the membrane, limits the effect of WSS on the EC-SMC interactions, and the effect is more similar to the that of the EC-EC interactions. The limited EC-SMC interactions found in the 2-side model resemble the limited interactions found *in vivo* in larger arteries, compared to the higher amount of MEGJ found in the 1-side model, which resembles the *in vivo* interactions in smaller arteries.

Thorough Inspection of the Z slices of the confocal images confirmed that the SMC and the endothelial layers remained as two separate monolayers on the opposites sides of the membrane in the 2-side model without migration of cells into the membrane or as 2 layers one on top of the other in the 1-side model. The distance between the two cell types in the 2-side model was measured to be roughly 20 µm. Although this is a large distance between EC and SMC, gap junctions have been reported to occur between the two cell types through pores present in the thin membranes [6], [26], [30]. In the 1-side model, the EC form a separate layer on top of the SMC and the cells do not migrate into the underlying SMC layer; hence the two cell types remain as two separate monolayers as well, though without a detectable distance.

In order to analyze the EC-Matrix interactions, we examined the FAK morphology of EC. In the 1-side model, the EC attached to the SMC directly and to the ECM produced by the SMC. This ECM differed from the fibronectin coated membrane that served as the attachment matrix to the EC in the EC monolayer and 2-side models. Thus the limited FAK stain (Fig. 3i-B) and reduced amount of FAK in the 1-side model (Fig. 4) might originate in the different FA complexes used to attach to the different matrices. Our finding is congruent with the work of Wallace et al, where EC cultured directly over SMC lacked focal adhesions, had reduced expression levels and less mRNA in several focal adhesion proteins. In their model, however, there was no difference in FAK expression between EC cultured on fibronectin coated plastic and EC cultured on SMC. They found that EC was mainly attached to SMC via α5β1 integrin whereas attachment to plastic involved both α5β1 and αvβ3. This difference in focal adhesion composition resulted in a different signaling cascade that drives different functions of the cell when exposed to WSS [27].

On the other hand, in a 2-side model as compared to the EC monolayer, an increase in the total amount of FAK was found in all WSS exposure modes (Fig. 4). This is similar to the finding reported by Wang et al where there was an increase the number of adherent ECs, and an increase of the total focal adhesion area (as measured using paxillin) in EC [29].

Following exposure to physiological WSS, the FAK stain became stronger and is easier to detect, showing an increase in the EC-matrix interaction. It is important to notice that this increase is probably not due to new formation of FAK but rather the recruitment and growth of existing FAK to the growing focal adhesion complexes. The antibody used for the FAK staining was a phosphorylated Y397 FAK (Abcam), suggesting that the increased stain was induced by increased phosphorylation of the FAK. Such phosphorylation was found in previous studies shortly after exposure to physiological WSS, and was accompanied by an increased amount of FAK at the focal adhesion sites [37], [38].

Our finding of an increase in the amount of FAK runs counter findings reported by Davies et al, where no differences in the total adhesion area of EC to the matrix were found following the application of WSS [39]. This discrepancy can be attributed to the quantification methods. Davies et al quantified the area of the adhesion site using digitized analysis of cells images, whereas we examined the images of a stained single protein in the adhesion complex. The increase we observed was due to a change in the protein complex. By contrast, in Davies’ work, no change in this complex was described. Moreover, our method may have been more sensitive in assessing the adhesion plaques. However, Davies et al noticed the same increase in the size of the focal adhesion complex as we did [39].

In the 1-side model (Fig. 3ii) following exposure to 12 dyne/cm^2^, the amount of FAK plaques increased as well as the size of each plaque, resulting with an increased amount of FAK (Fig. 4). This increase was equivalent to the amount of FAK in the EC monolayer model following exposure to 60 min of WSS. This could be due to the formation of new focal adhesions subsequent to WSS exposure, or perhaps to the formation of stronger attachments of the cells to the matrix that prevented detachment of the cells, or alternatively to phosphorylation of previously non-activated FAK in the focal adhesion complex. This phosphorylation could indicate the activation of mechano-transduction paths inside the cells.

The EC-EC interactions were analyzed in terms of the morphology and amount of the adherens junction VE-Cadherin. In the 2-side and 1-side models the staining of the VE-Cadherin was thicker, suggesting that the adherens junctions between the cells do not seal the cells to each other, but rather allow for a small gap (2–3 µm) between the endothelial cells. This could indicate a higher permeability level of the monolayer, suggesting breaks in the tight junctions or even a leaky endothelium [17]. This is in line with the quantification of the lower amount of VE-Cadherin expression in the co-culture models, indicating lower connectivity between the endothelial cells. This suggests that the presence of SMC can lead to a more inflammatory phenotype of the HUVEC. A similar phenotype can be found *in vivo* in areas of disturbed flow and inflamed endothelium, where VE-Cadherin expression is weak and discontinuous; however, it is significantly higher and continuous where the flow is laminar. Moreover, the areas of disturbed flow *in vivo* have been shown to be correlated with high permeability to plasma protein and cholesterol, which highlight the contribution of VE-Cadherin to intercellular widening, and the high permeability to macromolecules in these areas [8], [12]. In addition, Lavender *et al* found a decrease in PECAM-1 staining in a 1-side model as compared to EC monolayer model which was related directly to the EC-SMC interactions. Since PECAM-1 and VE-Cadherin are both located at the contacts of EC-EC, this a reduction is consistent with our findings [20].

Following the application of physiological WSS the VE-Cadherin stain ceased to be continuously distributed at the periphery of the cells and appeared in clusters at the cell borders. This was consistent in all 3 models; however, in the 1-side model the effect seemed weaker (Fig. 5C) and the discontinuity was only significant after longer exposure to WSS (i.e. 60 min). A discontinuity in VE-Cadherin staining of this type was found *in vitro* previously, following short exposure to disturbed pulsatile flow or following long exposure to reciprocating flow and physiological WSS [8], [11]. This change was not accompanied by a change in VE-Cadherin expression [8]–[10]; however there was a change in the cellular distribution of VE-Cadherin within the cell, allowing for cell alignment while still attached to each other [10]. Crucially, VE-Cadherin expression did not change within the models following exposure to the different WSS; hence the difference between the co-culture models and the EC monolayer model was still present following exposure to WSS.

Following exposure to 4 dyne/cm^2^ the discontinuity was not as strong as after exposure to 12 dyne/cm^2^ in all 3 models; however, in both co-culture models this change was not significant compared to the same model in static conditions. This suggests that the EC-SMC interactions might change the EC response to WSS and the alignment sequence of the cells. In addition, the change in the focal adhesion composition could possibly be attributed to the reduced amount of fenestration in the stains of the VE-Cadherin following exposure to 12 dyne/cm^2^ in the 1-side model (Fig. 5A–E) compared to the other 2 models.

Moreover, WSS was found to induce FAK phosphorylation and to increase the trans-electrical resistance of EC monolayer, suggesting that FAK may play a role in EC permeability [40]. This is supported by a recent finding where inhibition of FAK activity resulted in a leaky vessel, abnormal VE-Cadherin distribution and reduced VE-Cadherin phosphorylation. On the other hand, activation of FAK was related to increased endothelium barrier properties [41]. These data suggest that FAK is important in the control of cell-cell junctions and the barrier properties of the EC monolayer. It also provides a possible mechanism for the reduced expression of VE-Cadherin in the 1-side model and the increased thickness of the VE-Cadherin stain in this model.

Previous studies have shown that Cx37 is up-regulated when exposed to physiological WSS whereas Cx43 is up-regulated following exposure to pathological WSS, and hence exhibits different response to WSS stimuli of the different connexins, with independent regulation mechanisms [35]. Johnson and Nerem used a model of SMC in collagen gel and found an up-regulation in Cx37 expression in the presence of SMC which was even higher following exposure to WSS. In the case of Cx43, there was no protein expression in the EC monoculture either with or without flow. By contrast, in the presence of SMC there was high expression of the protein, which was down-regulated by the flow. These results demonstrate that the presence of SMC increases the expression of connexins in EC, which is probably due to the role of the connexins in MEGJ [35]. In our system, the amount of both Cx43 and Cx37 increased in the presence of SMC prior to exposure to WSS, suggesting that the excess amount of connexins was used to form MEGJ with the SMC. The amount of Cx43 was significantly higher in the 1-side model, which proves that the introduction of the membrane, which increases the distance between the cells, creates a barrier between the two cell types; however, such a difference was not found with Cx37.

The effect of exposure to WSS on the EC monolayer model was a 1.5 fold elevation in the amount of connexins in all WSS modes (partly for Cx37 in the case of a WSS of 12 dyne/cm^2^). Since the exposure time to WSS was only 30 min, this change is likely to correspond to the transient change found following the onset of the flow [42]. On the other hand, in the presence of SMC, with MEGJ formed, the exposure to a WSS of 12 dyne/cm^2^ did not induce any change in the amount of connexins. A possible explanation is that the higher amount of connexins from the MEGJ needs a longer exposure to WSS prior for a change to occur or that following exposure of the WSS the change in MEGJ requires longer exposure to WSS compared to EEGJ. A possible reason is that the connexins may move from MEGJ to EEGJ as an immediate response, before any change in the total amount of the protein takes place. Moreover, the amount of Cx37 in the EC monolayer following exposure to WSS of 12 dyne/cm^2^ appeared to have been equivalent to that found in the co-culture models without the WSS stimulation. It is important to note that in the EC monolayer model, Cx37 and Cx43 form gap junctions between the EC; thus differences in amount are indicative of the connectivity and communication of EC-EC (i.e. EEGJ).

Subsequent to a WSS of 4 dyne/cm^2^ the response of both co-culture models was consistent with what has been reported in previous works. The elevation of Cx43 expression was higher in the 2-side model than in the 1-side model; however it was the highest in the EC monolayer model. On the other hand, although Cx37 expression was down-regulated in the co-culture models, and was the lowest in the 1-side models, it was elevated compared to the control in the EC monolayer model. These changes in the magnitudes of the response show that the response of the 2-side model had more similarities with the EC monolayer response. This suggests that the EC-SMC interactions reduce the response to WSS; however, this was more prominent when there was no distance between the two cell types caused by the membrane between the cells in the 2-side model.

In our system we found that the presence of SMC changes the response of the cells to flow. However, the presence of a membrane between the cells apparently reduces the effect of the presence of SMC in both the connexins and the VE-Cadherin response. This change could not be ascribed to the changes in the amount of contact between the cells, since the change in the amount of Cx43 between the models was not notably high. On the other hand, the membrane found between the two cell types in the 2-side model increases the distance between the cells, so that the diffusion time of the secondary messengers’ increases, hence limiting or delaying the response of the cells. In addition, this change could be due to the different focal adhesion complex formed by the EC on the SMC layer as compared to the FA complex on the membrane.

*In vivo*, the presence of MEGJ has been demonstrated in different animal models and different vascular sites; however, the frequency of MEGJ increases with decreasing vessel size [43]–[45]. Furthermore, their presence in some large arteries is not considered proven [46], [47]. In addition, the MEGJ function is more important in resistance arteries rather than in conduit vessels because of their participation in mediated endothelium dependent hyperpolarization [48]. Taken together, these observations suggest that the effect of WSS on the EC-SMC interactions through the MEGJ is an important factor in vascular functionality; however, the differences in the cellular interactions in the different models simulate the *in vivo* interaction found in different types of blood vessels; i.e. small arterioles would be better demonstrated using the 1-side model, whereas larger arteries would be better demonstrated using the 2-side model.

## Conclusion

The data presented in this study demonstrate the effect of WSS on the EC-matrix, EC-EC and EC-SMC interactions. This was studied by measuring the changes in FAK, endothelial MEGJ and adherens junction proteins in response to WSS in two co-culture models and a control model of the EC monolayer. This is the first time that a reduction in the EC-EC interactions has been found *in vitro* in the presence of SMC, in both static conditions and following exposure to WSS, as demonstrated by the amount of VE-Cadherin. This was accompanied by increased distance between the endothelial cells, which suggest a less connected and more permeable EC monolayer that resembles the inflammatory endothelium *in vivo*. In addition, the amount of Cx37 and Cx43 increased in the presence of SMC, indicating the formation of direct EC-SMC interactions through the formation of MEGJ. Following exposure to both physiological and low WSS, EC-EC communication through EEGJ increased in both the Cx37 and the Cx43 in the EC monolayer model. The EC-SMC interactions, however, did not vary following exposure to physiological WSS. Low WSS, on the other hand, increased EC-SMC interactions through Cx43 expression, whereas it reduced EC-SMC interactions through Cx37. The magnitude of this response varied between the different co-culture models, suggesting that the distance introduced by the membrane in the 2-side model reduces the effect of WSS on the EC-SMC interactions and thus the response in the 2-side model was more similar to the EC-EC connexin interactions. These results show that EC-SMC interactions affect EC phenotype as well as the response of the cells to WSS and that the interactions formed between the cells depends on the distance between them. Altogether, this highlights the importance of cellular interactions to vascular wall functionality, and the importance of the interactions formed when simulating different types of blood vessels.
